# Conducting Polymer-Based Gel Materials: Synthesis, Morphology, Thermal Properties, and Applications in Supercapacitors

**DOI:** 10.3390/gels10090553

**Published:** 2024-08-26

**Authors:** Mohammad Mizanur Rahman Khan, Nilave Chakraborty

**Affiliations:** 1 Department of Mechanical Engineering, Gachon University-1342, Seongnam-daero, Sujeong-gu, Seongnam-si 13120, Gyeonggi-do, Republic of Korea; 2 Department of Chemistry, University of Utah, 315 South 1400 East, Salt Lake City, UT 84112-0850, USA; utah.chem@gmail.com

**Keywords:** conducting polymers, gels, synthesis, morphology, electrode materials, electrochemical performance, supercapacitors

## Abstract

Despite the numerous ongoing research studies in the area of conducting polymer-based electrode materials for supercapacitors, the implementation has been inadequate for commercialization. Further understanding is required for the design and synthesis of suitable materials like conducting polymer-based gels as electrode materials for supercapacitor applications. Among the polymers, conductive polymer gels (CPGs) have generated great curiosity for their use as supercapacitors, owing to their attractive qualities like integrated 3D porous nanostructures, softness features, very good conductivity, greater pseudo capacitance, and environmental friendliness. In this review, we describe the current progress on the synthesis of CPGs for supercapacitor applications along with their morphological behaviors and thermal properties. We clearly explain the synthesis approaches and related phenomena, including electrochemical approaches for supercapacitors, especially their potential applications as supercapacitors based on these materials. Focus is also given to the recent advances of CPG-based electrodes for supercapacitors, and the electrochemical performances of CP-based promising composites with CNT, graphene oxides, and metal oxides is discussed. This review may provide an extensive reference for forthcoming insights into CPG-based supercapacitors for large-scale applications.

## 1. Introduction

There is now a global demand to develop sustainable energy sources to overcome the problems of climate change, reduce environmental pollution, and so on [1]. Thus, it is necessary to discover low-cost and environment-friendly energy storage materials to employ the numerous energy resources. The construction of energy storage devices, like batteries, and supercapacitors from suitable materials, like conducting polymers, may provide an interesting focus at this time. Among the energy storage devices, supercapacitors have generated meaningful interest in the scientific community, owing to their superb characteristics, including fast charge–discharge capability, low cost, good specific power, and required safety [2,3]. Supercapacitors are also able to provide possible ways to achieve the growing demand for power needed in the energy sectors [4]. For example, supercapacitors have potential applications in electronic devices [5,6], hybrid electric vehicles [7,8], and so on. Further, they can suitably satisfy the energy gap between typical dielectric capacitors because of their elevated energy storage ability [1]. Such characteristics of supercapacitors provide the greatest potential in the field of energy storage devices [9]. Though supercapacitors have a lot of potential, they also have limitations for commercialization due to their low specific energy [10,11]. Therefore, it is essential to develop potential electrode materials to improve the specific energy of supercapacitors. In this domain, researchers have put substantial effort into fabricating specific energy three-dimensional (3D) supercapacitors using various materials, including polymers with porous structures such as sponges [12,13], hydrogels [14,15], aerogels [16,17], etc.

Among the numerous materials, commercially available supercapacitors are usually fabricated using various conductive additive materials and polymer binders [18]. However, the use of such additives and binders reduces the prepared electrodes’ electrochemical performance. Thus, it is necessary to prepare binder- and additive-free electrode materials to achieve the superb performance of the supercapacitors. To meet such requirements, conducting polymer gel or hydrogel may be an appealing material for prospective energy storage purposes [19,20]. In this context, the formation of conductive polymer gel or hydrogel for energy storage devices like supercapacitors is considered very promising because of their 3D cross-linked networks and hydrophilic polymer structures. These structural features of conducting polymer gel or hydrogel are responsible for their better mechanical properties as well as for the development of multifunctional features in the electrode materials.

Thus, conductive polymers (CPs) have generated much consideration by researchers owing to their controllable electrical conductivity and ease of synthesis [21,22]. In addition, conducting polymers with unique nanoscale dimensions can exhibit remarkable physical and chemical properties, which are useful for practical applications [23,24]. Such features of CPs have a positive impact on the advancement of nanoscience and nanotechnology. For instance, CPs with 0D, 1D, and 2D nanoscale structures have already been applied in various fields such as electronics, conversion devices, sensors, and energy storage systems [25,26,27]. However, CP synthesis with a suitable morphological form and appropriate thermal stability for the applications of energy storage devices like supercapacitors is still challenging. Recently, conductive polymer gels (CPGs) comprising 3D networked forms were synthesized with the intention of cross-linking the conjugated polymer processions for high electrochemical activity and device applications [28,29]. It is known that CPGs exhibit the distinctive characteristics of gels, as they possess dilute cross-linked arrangements and show no flow at the steady-state stage. These structural features of CPGs make them advantageous in the areas of flexibility, ionic conductivity, and electrochemical activity [22,30]. Owing to these advantages of CPGs, they have been widely used in diverse areas, including responsive devices and supercapacitors.

The current review is aimed to stipulate the modern developments in CPG material fabrications for supercapacitor applications. The types of supercapacitors and electrochemical performance assessment approaches are also discussed. Further, we have clarified here the recent research work on the numerous synthesis techniques and their significance, as well as the morphology and thermal properties of these types of CPGs as electrode materials. The recent progress on using conducting polymer-based potential composites for electrochemical performance is also discussed in this review.

## 2. Synthesis of Conducting Polymers, Morphology, and Thermal Properties in Gel Forms

### 2.1. Typical Conducting Polymers

**Figure 1:**
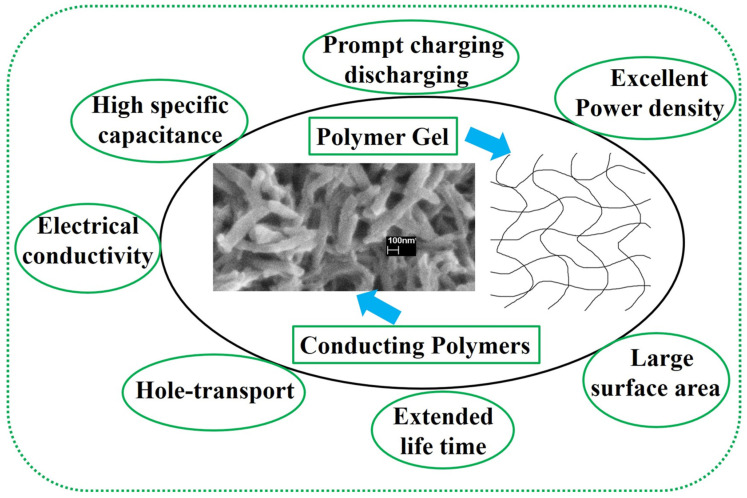
Schematic illustration of the basic properties of a conducting polymer gel for supercapacitor applications.

**Figure 2:**
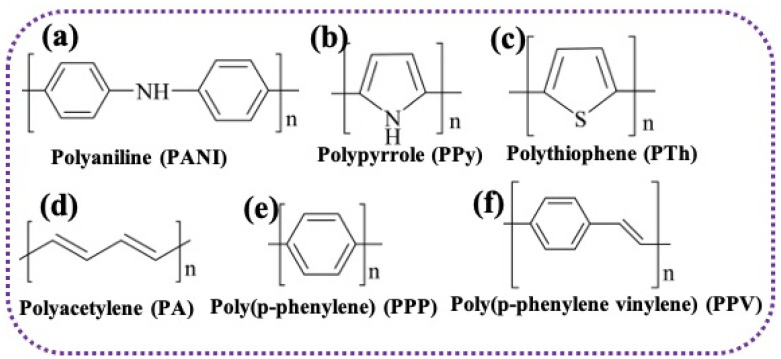
(**a**–**f**) Molecular structure of popular conducting polymers.

Conducting polymers (CPs), including polyaniline (PANI, polypyrrole (PTh), and polythiophene (PTh)), are typically well-known for their easy synthesis approach, environmental stability, and various promising applications such as electronic devices, light-emitting diodes, and energy storage materials. Some basic properties of CPs are displayed in Figure 1, and the molecular structure of such CPs is presented in Figure 2. Among the numerous forms of CPs such as nanofiber, nanotube, nanosheet, etc., the polymers in the gel form or hydrogel form play a significant role in their potential applications including in supercapacitors. Concerning the synthesis, structure, and properties of such conducting polymers gels/hydrogels (CPHs), these are reviewed below:

### 2.2. Synthesis Approaches

**Figure 3:**
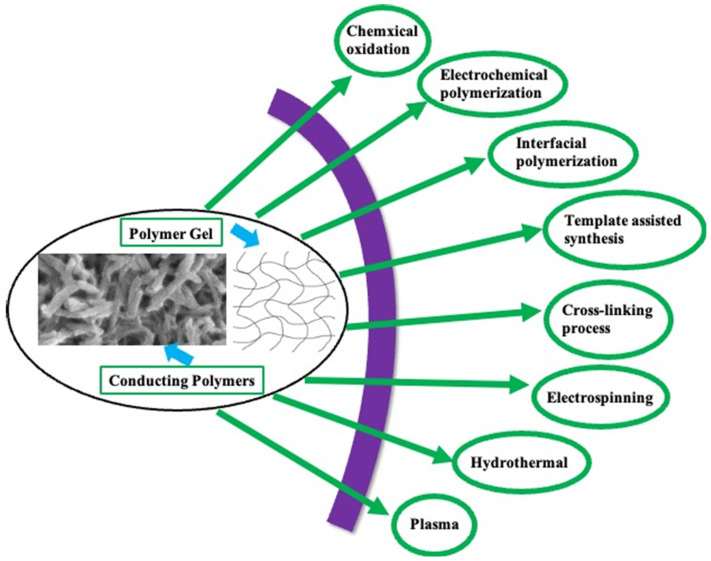
Schematic illustration of the various synthesis approaches of conducting polymers gels.

The most commonly known synthesis techniques for CPs and their gel forms are in situ chemical oxidation, electrochemical polymerization, interfacial polymerization, template-assisted synthesis, and the cross-linking process as illustrated in Figure 3 (Table 1). Such synthesis approaches are reviewed in this section.

#### 2.2.1. Synthesis of Conducting Polymers (CPs)

Conducting polymers (CPs) are very useful for the fabrication of commercially feasible supercapacitive materials [12,31,32]. Polyacetylene, which was the first CP, was reported in Reference [33]. The very common types of obtained CPs using different synthesis approaches include polyaniline [34], polypyrrole, [35,36], polythiophene [37], etc. These polymers are usually synthesized via chemical or electrochemical approaches [38,39]. The chemical polymerization technique is the more feasible method where the reaction occurs with the monomer using an oxidizing agent in a suitable acidic condition. The other method, electrochemical polymerization, typically involves a three-electrode arrangement. The cell comprises a working electrode (for example, metals, conductive oxide), a reference electrode (for example, Ag/Ag), and a counter electrode (for example, Pt, Au) in a monomer solution and an electrolyte in a solvent.

**Table 1:** Polymerization approach of the conducting polymer synthesis; their advantages, disadvantages, and significance are listed.

Polymerization Approach	Advantages	Disadvantages	Significance	Refs.
In situ chemical oxidative	Feasible method, low cost, scalability	Complex processing	Straightforward approach	[38,39,40]
Electrochemical	Feasible, porosity management	Costly, complex synthesis, scalability difficulty	Autocatalytic	[39,41]
Interfacial	Feasible, convenient product separation	Time consuming	Controllable polymer structure	[42,43]
Cross-linking	Well-suited with a biological approach	Post cross-linking, low mechanical properties	Stable polymer structure	[44,45,46,47]
Template assisted	Polymer formation in gel forms	Both conductive and nonconductive components formation	Better solubility of monomer and template	[48,49,50]
Electrospinning	Simple, versatile, cost-efficient	Low solubility and brittleness of products	Depend on operating parameters	[51]
Hydrothermal	Simple, inexpensive	Low tensile strength	Achieve better polymer crystallinity	[52]
Plasma	Polymer formation in film forms, product without typical contamination	Complication of plasma processes	Solvent-free conditions, room temperature synthesis	[53]

#### 2.2.2. Synthesis of Conducting Polymer Hydrogels (CPH)

Hydrogels have a network matrix with highly augmented and intertwined polymers that comprise of substantial amount of water (90–95%) in their arrangement. Such gels also have a spatially arranged cross-linked sequence linkage constituted by a hydrophilic polymer chain. Due to their special structural arrangement, CPHs possess some promising properties like softness, porosity, and a high surface area [54,55]. Further, they possess mixed electronic and ionic conductivity and transformation between conducting–insulating forms in CPs. The most familiar conductive materials of CPHs are PEDOT, PANI, PPy, etc. [56,57,58].

**Figure 4:**
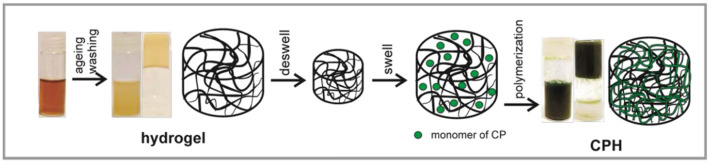
Schematic illustration for the synthesis of a CPH comprised of an accompanying polymer and CPs [70]. Copyright 2019, MDPI.

The CPH synthesis can be performed using numerous polymers as reported in References [58,59]. The utilization of such polymers for the fabrication of CPHs has advantages as well as disadvantages. Up to now, several methods have been conducted for the synthesis of CPHs [60,61,62]. Among the approaches, the most reliable one is the mixing of the hydrogel constituent with the CPs. Another method is also preferred where the polymerization reaction of CP monomers occurs in the form of a hydrogel. In this context, the very well-known technique used is the synthesis of a hydrogel with a supportive polymer, which then can generate a matrix for the preparation of a CP [63]. Such an idea is given in Figure 4, where it can be seen that CPH consisting of CP is constructed in a matrix generated from water-soluble polymers (Figure 4). The CP solution and the deswelled hydrogel are mixed which absorbs monomers until they reswell to the typical volume. Afterwards, the reswelled hydrogel is dipped in a solution of oxidative initiators and dopants, which ultimately allows for the in situ polymerization process of CPs. Finally, the CP is formed after meeting with the monomer. This synthesis approach is known as interfacial polymerization (Figure 4). Such a synthesis technique is used to obtain the CPHs, where the CP function is satisfied with the PANI, PPy, and sometimes with PEDOT [64,65,66,67,68,69].

To synthesize PANI and PPy, the researchers also used the modified interfacial polymerization technique [70,71]. By the addition of CP powders into the reaction mixture system, CPHs can also be synthesized which is the basis for obtaining a hydrogel. Using such a synthesis procedure, the derivatives of PANI, PPy, and PEDOT can be obtained [71,72,73]. The significance of the specific functions of these gel materials is firmly correlated with their particular uses. These CPHs possess nontoxic properties and are well-suited with easy handling for several applications [74,75]. Moreover, the potential properties of CPHs offer their applications in various attractive areas like energy storage (for instance, supercapacitors).

**Figure 5:**
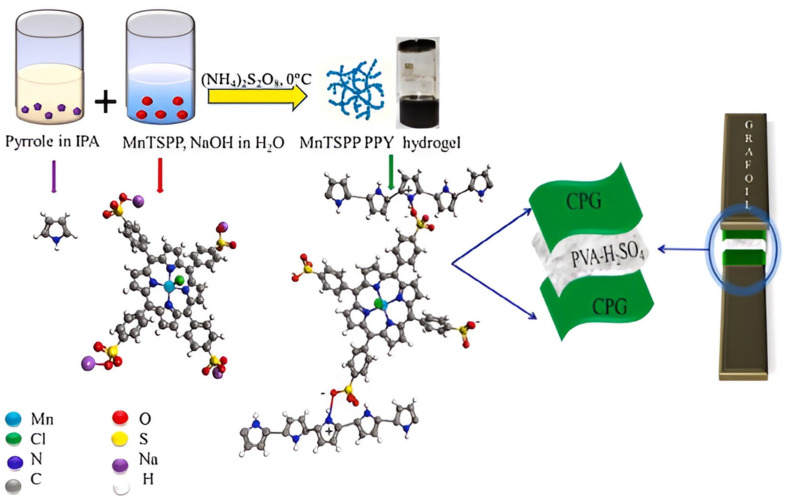
Schematic illustration of the synthesis of the PPy hydrogel doped with MnTSPP. Reproduced with permission from Ref. [1]. Copyright 2022, Elsevier.

A different synthesis process of conducting polymer hydrogel (CPH) is described below. Because of the interest in the synthesis of CPH, researchers have developed many synthetic approaches for their fabrication such as (i) the polymerization of the monomer in non-conductive frameworks and templates by in situ chemical or electrochemical means; (ii) adding cross-linking agents, like phytic acid, to the polymer backbone; and (iii) co-polymerizing the monomers of a conductive polymer and non-conductive one [61,76,77]. For instance, 3D polyaniline/phytic acid supramolecular hydrogels were synthesized by the electrochemical polymerization technique [65]. To demonstrate the versatility of phytic acid for the synthesis of 3D CPHs, Shi et al. [78] prepared a conducting PPy hydrogel by an interfacial polymerization method where the synthesis was complemented by an aqueous/organic biphasic line. For the synthesis of PPy hydrogels using 5, 10, 15, 20-tetrakis (4-sulfonatophenyl)−21H, 23H-porphine manganese (III) chloride (MnTSPP) as a dopant and cross-linker was used by Das et al. [67]. The supramolecular synthesis approach of the PPy hydrogel, employing MnTSPP as a cross-linker and dopant, is schematically represented in Figure 5.

### 2.3. Other Synthesis Strategies for the Formation of Conducting Polymer Gels (CPGs)

#### 2.3.1. Synthesis of Cross-Linked CPGs

**Figure 6:**
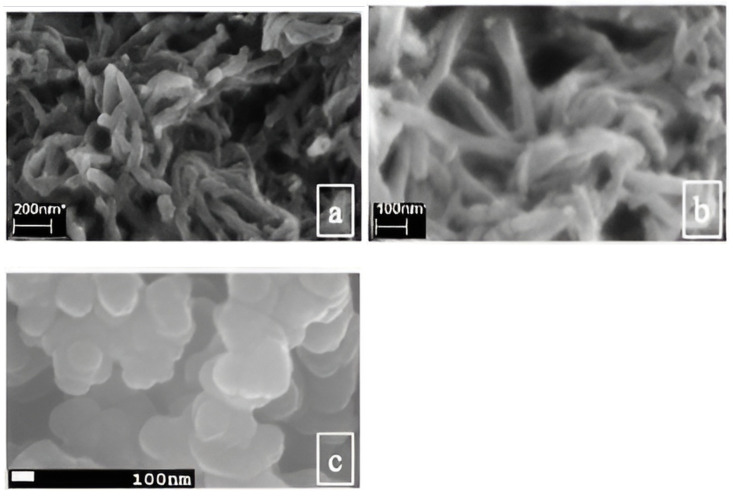
(**a**) SEM micrographs of the PANI nanofibers. (**b**,**c**) FESEM micrographs of diverse nanostructured PPy gels obtained through changing dopants.

The synthesis of CPs including PANI, PPy, PTh, etc., in various forms like fiber, gel, etc., has already been reported with the construction of their 3D nanostructured networks using template-guided methods which can be soft- or hard-template methods [48,49]. These approaches usually provide polymer in gel form but possess some limitations including the formation of both conductive and nonconductive sections. For example, in a recent study, it has been reported that phytic acid and copper phthalocyanine-3,4′,4′′,4′′′-tetrasulfonic acid tetrasodium salt (CuPcTs) molecules which have multiple functional groups, can cross-link with the CP chains; ultimately, the formation of the CPGs with 3D networked forms free of nonconductive sections [50,60,79,80]. The mechanism of such a formation involves the reaction of these types of molecules with the CP chain through protonating nitrogen groups as well as by electrostatic interactions, therefore acting as dopants and cross-linkers. Thus, such dopant molecules are cross-linked with the CPGs which are able to exhibit better electrical and ionic conductivities. These performances are connected with the morphology of the PANI (Figure 6a).

It is possible to change the chemical and physical properties of the dopant molecule cross-linked CPGs. By altering the cross-linker employed in the synthesis process, it is feasible to modify the dopant molecule cross-linked CPGs’ chemical and physical properties. On the other hand, indigo carmine and indigo carmine dehydrate were used to attain PPy gels with a granular nanostructure and a 1D nanostructure that was “necklace-like” [79]. A similar morphology of the nanostructured PPy is displayed in Figure 6b,c. The electrical and electrochemical characteristics of the generated CPGs are significantly influenced by these diverse microstructures, which is significant for their applications in supercapacitors. Furthermore, the shape and thermal characteristics of CPGs can be further regulated by maintaining the synthesis parameters, including the temperature, solvent ratio, and precursor ratio. Thus, the microstructural characteristics of PPy gels can be adjusted by using various kinds of organic solvents and varying the ratio of monomers to cross-linkers [81].

#### 2.3.2. Double Network Structured Gel Synthesis Using CPGs

To obtain multifunctional gels, there is the potential to design and fabricate the gel structure with a diffusing double network. These types of gels can receive the benefits of each element and provide enthusing unique characters owing to the synergistic effect between two polymeric structures [82,83]. In this context, because of the progressively porous structure, the CPGs may be the perfect matrix to introduce a second gel network into this situation to create an interpenetrating double-networked hybrid gel. There are two different approaches to synthesizing double-network structured hybrid gels depending on CPGs. First, one of the gel structures is built, serving as a supporting matrix for the second polymeric network’s in situ polymerization. This is a two-step polymerization procedure. For instance, a conducting polymer of PANI and PPy-based hybrid gels such as poly (N-isopropylacrylamide) (PNIPAM)/PANI and PNIPAM/PPy was previously synthesized utilizing this technique [84]. The other method involves the one-step approach in which two polymers are subjected to the polymerization process and cross-linked in one starting reagent solution [85].

#### 2.3.3. Hybrid Gels Synthesis Based on CPGs

To attain effective features such as exceptional biocompatibility, a large surface area, and switchable conductivity, CPGs can play a vital role by acting as a supporting matrix for the immobilization of functional particles [86]. The hybrid gel can be synthesized using functional particles, including inorganic and biomolecules by loading them onto the surface of a progenerated CPG network by a straightforward solution-based deposition [87]. To create a chemical link between the functional particles and the CPG network, cross-linking agents, for example, glutaraldehyde, can be further introduced. In this way, the linking strength can be improved. In the hybrid gel formed following this way, CPGs play the role of a supporting matrix that immobilizes these functional particles within an electrochemical electrolyte and mediates the transfer of functional particles to substrates.

Therefore, relating to the synthesis of CPGs, such polymers can provide a unique era towards the development of flexible energy storage devices as they possess a superior flexibility and exceptional electrochemical performance. Despite such potential, including a better electrical conductivity, CPGs generally suffer from a poor mechanical strength and integrity, limiting their practical applications. Although appreciable efforts have been made by the researchers to stabilize and overcome the limitations, further research is needed focusing on the design and synthesis to improve the performances of conducting CPGs-based electrode materials for supercapacitors.

### 2.4. Morphology

**Figure 7:**
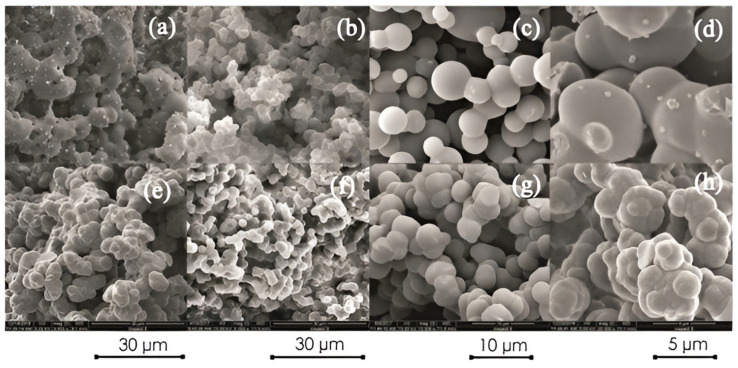
(**a**–**h**) SEM micrographs with various magnifications of aero-sponge structure composites synthesized from distinctive hydrogels [70]. Copyright 2019, MDPI.

The morphological features are also discussed along with the synthesis process of CPGs in the earlier sections. CPGs like PANI and PPy are obtained with interesting morphological features synthesized by different approaches like the interfacial polymerization technique [70,71,88]. For example, aero-sponge-like materials (Figure 7) can be obtained if liquid is substituted with gas (Figure 7a–h). In this case, the created pores remain the same and the porous morphology is not demolished. Such porous structures can be appreciated in Figure 7a–h for hydrogel and aero-sponge-like materials. Thus, SEM images are presented in Figure 7a–h of hydrogel and aero-sponge-like materials showing their unique morphology, such as being porous, and their highly developed surface area, which are useful features for several applications.

As can be seen from Figure 7, CPs have numerous morphological features. The CPs can also be prepared in the form of a fiber, gel, rod, tube, etc. For example, PANI can be formed in 1D structures in nanotubes, nanofibers, and nanorods morphologies [89,90,91]; planar 2D objects, e.g., ribbons, nanobelts, and nanoplates [90,92]; and 3D particles such as granules, microspheres, and nanospheres [93,94]. These types of morphological features promote CPs for the prospect of enriched functioning for potential applications including supercapacitors [95]. On the other hand, in most of the cases, the obtained CPs exhibited an aggregated, irregular morphological topography which is a limitation for their large-scale applications in different areas like supercapacitors. However, more investigations are required to obtain the appropriate morphology of CPGs for their successful application in supercapacitors.

### 2.5. Thermal Properties

The thermal stability of the conducting polymer gel is important because of its proper applications in various areas including electronics cooling, flexible actuators, supercapacitors, etc. In the case of the containment of moisture in the gel structure, they are prone to losing mechanical toughness as well as declines in thermal properties/stability. Moreover, because of the loss of moisture or loosely bounded water in the structure of the gel, some other excellent properties can also be lost such as electrical conductivity [96,97], self- repairability [98,99], etc., which may severely limit their applications in their respective fields. Thus, it is essential to improve the thermal stability of conducting polymer gel materials not only for energy storage applications like supercapacitors but also to present temperature-independent mechanical behavior and superb anti-freezing and anti-drying properties. To mitigate such issues, researchers are focusing on work improving their properties including their morphology and thermal stability. Further, a lot of work has already been performed by scientists, such as investigating anti-freezing properties, modifications of polymer networks, anti-drying properties, etc. [100,101]. Thus, the applications of CPG materials largely depend on their fabrication as well as on their unique properties including their thermal stability. Thus, for large-scale industrial applications, CPG materials must adapt to the environment and improve in thermal stability, along with presenting high temperature-independent mechanical behaviors, morphological behavior, and excellent anti-freezing properties. For these purposes, researchers have conducted much work, although it is a still challenging area of research. In the following section, the basics and design of supercapacitors, and the constructions of various supercapacitors which are employed to measure the electrochemical performance of materials will be discussed.

## 3. Basics of Supercapacitors

The ever-increasing demand for energy is propelling modern civilization toward utilizing clean and sustainable energy to diminish the economic and ecological drawbacks of using conventional energy sources, e.g., oil, gas, and coal. However, renewable and clean energy sources like solar and wind are intermittent and sometimes unpredictable due to regional weather conditions. For instance, solar panels may be ineffectual in cloudy weather, and wind turbines may be unproductive in calm weather. In addition, renewable energy sources could sometimes overload the system by producing excess energy. Therefore, it is essential to account for these shortcomings of renewable energy sources and ensure the proper balance between energy generation and demand. In this regard, energy storage technology is crucial for controlling the variable nature of renewable energy sources and supplying the required energy. Batteries, fuel cells, and supercapacitors are at the forefront of contemporary energy storage devices [102,103,104,105]. Among these various energy storage systems, supercapacitors have garnered extensive attention from the scientific research community due to their rapid charge–discharge ability, high cycle stability, quick burst power supply, longevity, broad range of operating temperatures, and applicability in various electronic devices and electric vehicles [106,107,108]. As supercapacitors have a high power density, they are used in applications that require a high and stable energy throughput, such as hybrid electric vehicles, computer chips, portable electronic devices, etc. [109,110,111].

**Figure 8:**
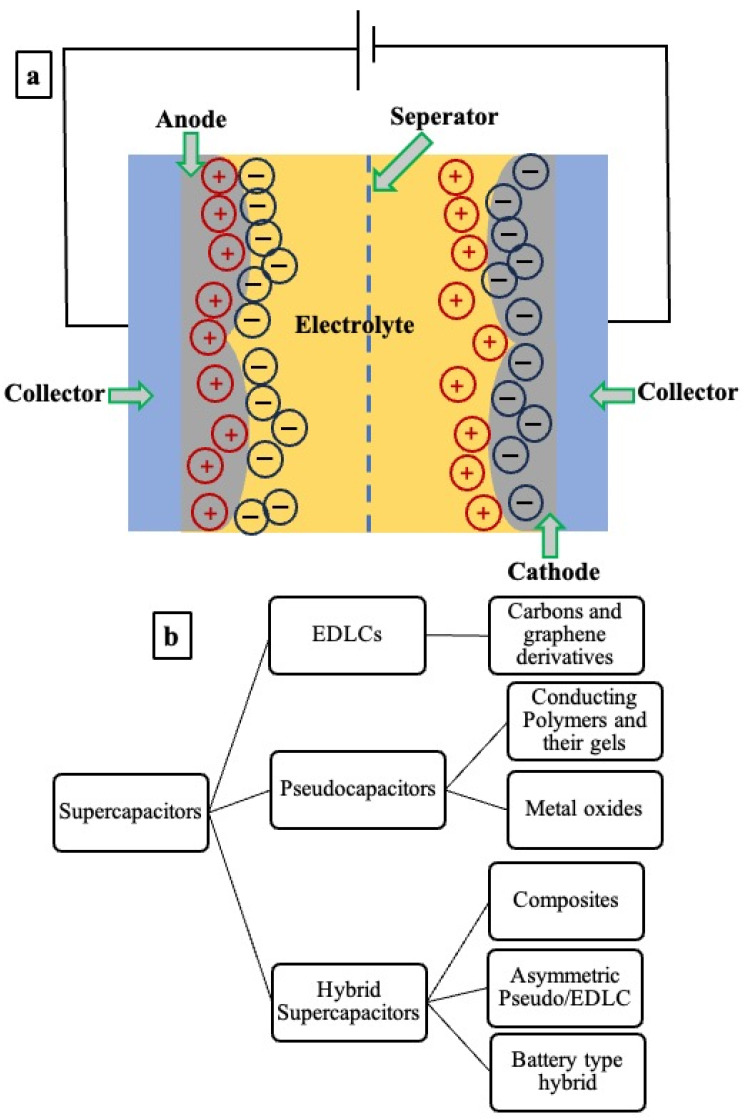
(**a**) Schematic representation of a conventional supercapacitor [124], and (**b**) the classification of supercapacitors [125,126,127].

Supercapacitors are primarily electrochemical cells comprising two electrodes (e.g., anode and cathode; in supercapacitors, the terms anode and cathode are commonly used even though energy may not be delivered via redox reactions [112] in contact with current collectors separated by an electrolyte-infused porous ion-conducting and electron-insulating membrane (Figure 8a).

The individual role of each component defines the overall performance of supercapacitors. For example, a current collector should have a good stability, high conductivity, and proper mechanical properties for high-performance supercapacitors [113,114,115]. In addition, an ion-permeable porous separator and an electrolyte with strong ionic conductivity are necessary for ionic charge transfer and electrochemical properties, respectively [116,117]. Moreover, electrodes, pivotal for converting and storing electrochemical energy, must have a good conductivity, high thermal and chemical stability, mechanical stability, wettability, corrosion resistance, and high surface area [118,119,120,121]. Therefore, the materials in the electrode are the deciding factor in achieving the superior performance of supercapacitors [122,123]. As a result, recent research has focused on electrode materials to increase the energy density to match that of batteries. This review will focus on using a conducting polymer gel as an electrode material to enhance supercapacitor performance.

### 3.1. Types of Supercapacitors

Supercapacitors are energy-storage devices that use an electrostatic charge separation process to store electrical energy. Based on the energy storage mechanism, superconductors can be made from different materials such as carbons and graphene derivatives, composites, conductive polymer and their gels, metal oxides, etc., and classified into three categories as shown in Figure 8b [125,126,127].

(i) Electric double layer capacitors (EDLCs);
(ii) Pseudocapacitors;
(iii) Hybrid supercapacitors.

The EDLCs, standard or conventional supercapacitors, include two symmetrically oriented identical carbon-based electrodes. They use the physical adsorption of charges at the electrode/electrolyte interface to store energy by forming an electric double layer. It is worth mentioning that supercapacitors are wet electrolytic capacitors that use ion-containing liquids to facilitate charge transport [111]. Pseudocapacitors use a combination of electrochemical doping and redox reactions to store energy. Although their energy storage density is higher than EDLCs, they suffer from slower charge–discharge rates. Last but not least, hybrid supercapacitors, living up to their name, combine the energy storage mechanisms of both EDLCs and pseudocapacitors. They are well suited for various energy storage applications because of their ability to balance the high energy-power density.

### 3.2. Charge Storage Mechanism

**Figure 9:**
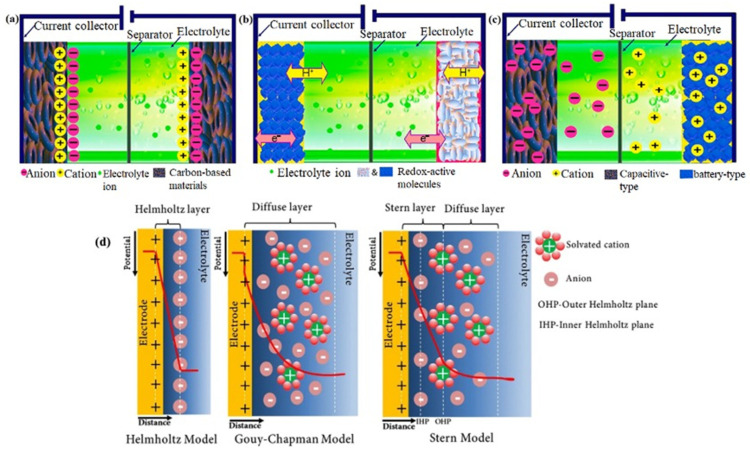
Schematic representation of (**a**) an EDLC, (**b**) a pseudocapacitor, and (**c**) a hybrid supercapacitor [1]. Reproduced with permission from Ref. [1]. Copyright 2022, Elsevier. (**d**) Schematic representation of EDLC structures: Helmholtz, Gouy and Chapman, and Stern models [137]. Reproduced with permission from Ref. [137]. Copyright 2020, Springer.

Supercapacitors have three ways of storing charge: electrostatic (Helmholtz double-layer and non-Faradaic), electrochemical (Faradaic), and a combination of both electrostatic and electrochemical (hybrid) charge storage mechanisms [111,128,129,130]. The Faradaic charge transfer process is characterized by fast and reversible oxidation–reduction (redox) reactions between electrodes and electrolytes. Conversely, non-Faradaic methods use physical processes to distribute charge on the electrode surface. As mentioned above, supercapacitors are classified based on their charge storage mechanism. Therefore, each class of supercapacitors has its unique charge storage mechanism, for example, non-faradaic (EDLCs), Faradaic (pseudocapacitors), and a combination of two (hybrid supercapacitors), as shown in Figure 9a–c [1,131,132,133].

#### 3.2.1. Electric Double Layer Capacitors (EDLCs)

EDLCs are the most common supercapacitor type and constitute the majority in the commercial market. The EDLC, first proposed by Helmholtz in 1853 [134], is a device where the immersion of an electrode into an electrolyte leads to a spontaneous charge organization at the interface of electrode/electrolyte, forming an electrical double-layer with one layer at the surface of the electrode and the other in the electrolyte [116,135]. Helmholtz’s concept considers rigid layers of charges from the solid electrodes counterbalancing each other [4,136], which was later modified by the diffuse model proposed by Gouy and Chapman. According to Gouy and Chapman, the opposing ionic charges gradually permeate into the bulk electrolyte rather than being firmly adhered to the electrode surface [4,136]. Stern further modified the diffuse model of EDLCs and combined the abovementioned models [126,128]. The Stern method has two layers: the Stern layer (inner, compact layer) and the diffuse layer [126,128,136]. In the Stern model, the total double-layer capacitance (*C_dl_*) is given by [126,128,136],

(1) $$\frac{1}{C_{dl}}=\frac{1}{C_{H}}+\frac{1}{C_{diff}\;\;}\;,$$

The schematic diagram of all three models (i.e., Helmholtz, Gouy and Chapman, and Stern) is presented in Figure 9d [137].

The source of double-layer capacitance is the potential dependence of the surface energy electrostatically stored at the capacitor electrodes’ interface [110]. This electrostatic buildup creates an electric double layer, storing charges without a chemical reaction [138]. Hence, in EDLCs, the energy is stored non-Faradaically without any electron exchange or redox reaction [110,126]. Consequently, there are no corresponding changes in chemical composition, which renders reversibility and high cycling stability (over 100,000 cycles) to EDLCs [139]. In addition, EDLCs offer a high power density and fast charging–discharging rate (in seconds) because of the rapid absorption and desorption of the ions at the electrode surface [125]. For these reasons, EDLCs find their use in places, including automotive systems, renewable energy storage, and portable electronic devices, where the long cycle life and capability to supply rapid bursts of energy are much needed [125].

The thickness of the Helmholtz layer (approximately 1 nm) and the electrodes’ huge surface area are crucial for achieving an extraordinarily high capacity [110,123]. The highly expanded surface area of the electrode augments EDLCs’ ability to store more charge, leading to a higher capacitance. It is common practice to enhance the electrodes’ surface area per unit mass or volume using porous electrodes to achieve a higher capacitance per unit mass or volume. In addition, a large porosity and an appropriate pore distribution of the electrode materials are crucial in determining EDLCs’ performance. Maximum double-layer capacitance can only be achieved when the pore and the ion size are very similar [140]. Carbon-based materials, such as activated carbon, carbon nanotubes, graphene, and others, are considered the most suitable electrode materials for EDLCs due to their large surface area, availability in different forms, tunable porosity structures, high conductivity, high thermal and chemical stability, compatibility and processability in composites, and low cost [135,141,142,143,144].

#### 3.2.2. Pseudocapacitors

**Figure 10:**
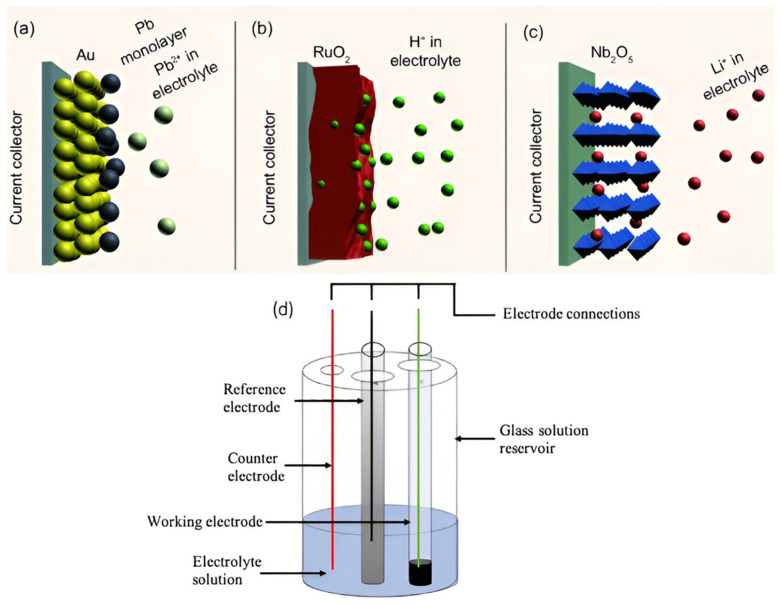
Various kinds of reversible redox mechanisms that induce pseudocapacitance: (**a**) underpotential deposition, (**b**) redox pseudocapacitance, and (**c**) intercalation pseudocapacitance [154]. Reproduced with permission from Ref. [154]. Copyright 2023, Elsevier. (**d**) Schematic representation of a three-electrode configuration.

The pseudocapacitor, also known as the Faraday capacitor, is another supercapacitor type designed to procure a high energy density to compete with batteries. However, they are much less commercially available than EDLCs. In pseudocapacitors, the capacitance arises on the surface and the bulk near the surface between the electrode materials and electrolyte ions via fast and reversible Faradaic redox reactions. These Faradaic reactions involve the passage of charge across the double layer, similar to the charging/discharging of batteries. Hence, pseudocapacitors’ operation principle is closer to batteries than supercapacitors [110,145]. Essentially, the capacitance in pseudocapacitors arises for thermodynamic reasons, expressed as the amount of charge accepted (∆*q*) and the change in potential (∆*V*), and it is the derivative C = $\frac{d\left(∆q\right)}{d\left(∆V\right)}$ that is referred to as pseudocapacitance [4,110,146]. Three primary forms of pseudocapacitance can be distinguished (Figure 10): (a) underpotential deposition, defined by the monolayer adsorption of metal ions on a different metal’s surface, (b) redox pseudocapacitance, which is realized by Faradaic reactions, and (c) intercalation pseudocapacitance, which is based on the intercalation of the electroactive species in the layer without changing the crystallographic phase [103,147]. Among these three, redox and intercalation pseudocapacitances are the most common in designing pseudocapacitors due to their high-speed charge/discharge rate without diffusion limitation, which creates an obvious distinction from batteries.

Typically, the specific capacitance and energy density of pseudocapacitors are orders of magnitude larger than those of EDLCs because pseudocapacitance occurs both inside and on the surface of the electrode. However, pseudocapacitors usually suffer from a relatively lower power density than EDLCs due to the involved reaction dynamics (i.e., non-Faradaic processes are faster than Faradaic ones) [145,148]. In addition, redox reactions occurring at the electrode result in mechanical degradation and volumetric changes in the electrode material because of the shrinking and swelling of the electrode material in the charge–discharge process. Therefore, pseudocapacitive electrode materials have a lower cycling stability and rate capability than EDLCs, limiting their further commercialization.

The degree of pseudocapacitance mostly depends on the type of electrodes and their surface area. Pseudocapacitive materials, such as metal oxides, especially transition metal oxides and conductive polymers, are commonly used to store charge in pseudocapacitors because of their extensive capacitance storage capabilities [118,134,144]. Metal oxides like ruthenium oxide (RuO_2_) or manganese dioxide (MnO_2_) have been exploited as electrode materials for pseudocapacitors because of their ability to undergo fast redox reactions. However, their conductivity is relatively low compared to the other materials usually used in pseudocapacitors [4,136,149]. On the other hand, conducting polymers possess a relatively high conductivity and capacitance [150]. Some examples of conducting polymers that are reported as electrode materials used for pseudocapacitors include PANI [151], PTh [37], PPy [152], poly(3,4-ethylenedioxythiophene) [153], etc. Recently, the gel form of the conducting polymers has become an attractive scaffold for developing pseudocapacitors [1].

#### 3.2.3. Hybrid Supercapacitors

Hybrid supercapacitors comprise a pseudocapacitive or battery-type electrode with an EDLC electrode [155,156]. As the name implies, hybrid supercapacitors combine the energy storage mechanisms from EDLCs (non-Faradaic) and pseudocapacitors (Faradaic) simultaneously to harness the advantages of both EDLCs and pseudocapacitors while mitigating their limitations [128]. This combination improves performance characteristics such as providing a high specific power and energy by enabling the rapid charge/discharge rates typical of EDLCs and additional energy storage through reversible Faradaic processes. Therefore, by combining Faradaic and non-Faradaic processes, hybrid supercapacitors can balance high power and energy densities without compromising cycle stability and affordable cost [157,158]. Hybrid supercapacitors can be constructed using three primary electrode materials, which are further distinguished based on their electrode materials: composites, asymmetric, and battery-type [128,149,159]. Composite electrodes are typically carbon-based materials with conducting polymers or metal oxides integrated into them as pseudocapacitive materials. The highly porous structure and large surface area of carbon-based materials facilitate capacitive double-layer charge by amplifying the interfacial interaction between pseudocapacitive materials and electrolytes. Additionally, the pseudocapacitive materials enhance the capacitance of composite electrodes through the Faradaic process [149]. Asymmetric hybrid capacitors employ two distinct electrode materials: a cathode is made up of pseudocapacitive materials (e.g., conducting polymers or metal oxides) and an anode is composed of carbon-based materials with a high surface area [160]. They merge non-Faradaic and Faradaic processes by coupling an EDLC electrode with a pseudocapacitive electrode. By doing so, they fill the gap between effective energy storage for extended periods and quick charge/discharge [149,154]. Like asymmetric hybrids, battery-type hybrid supercapacitors combine two distinct electrodes: a supercapacitor and a battery electrode. This combination blends the advantages of battery and supercapacitors in one capacitor, for example, a lithium-ion capacitor [161].

### 3.3. Assessment of Supercapacitive Performance

Supercapacitors’ performance can be analyzed by evaluating their performance-governing properties, such as specific capacitance, specific energy, specific power, and cycle stability [162,163].

#### 3.3.1. Specific Capacitance

A supercapacitor’s total capacitance is the reflection of the stored electrical charge *Q* at an operating voltage *V*, defined as

(2) $$C=\frac{Q}{V},$$

Equation (2) [1,2] is preferred when specifying a supercapacitor device’s total charge storage ability. To measure the charge storage ability of a supercapacitor material, a more intrinsic specific capacitance (Cs) is defined as

(3) $$\mathrm{Cs}=\frac{Q}{aV},$$

where “*a*” can be the mass (g), volume (cm^3^), or surface area (cm^2^) of the electrode material, and even the size (cm) of the electrode, and the corresponding specific capacitance (Cs) value is often known as the gravimetric (F/g), volumetric (F/cm^3^), areal (F/cm^2^), and linear (F/cm) capacitance, respectively [1,2]. Carbon-based materials can provide a capacitance of more than 100 F/g [164], while pseudocapacitive materials can deliver a much greater capacitance [165,166].

#### 3.3.2. Specific Energy

The specific energy (Es) is the quantity stored in a supercapacitor per unit mass of the capacitor, which is evaluated gravimetrically or volumetrically in Wh/kg or Wh/L to manifest the amount of energy stored. The specific energy can be expressed as follows:

(4) $$\mathrm{Es}=\frac{C_{cell}{\left(\Delta V\right)}^{2}}{2}\times \frac{10}{36},$$

where *C_cell_* is the cell capacitance (F) and ∆*V* is the IR drop-free voltage window (*V*) [1,122]. Equation (4) shows that the specific energy can be enhanced by varying the capacitance or the voltage. Generally, the capacitance (Cs) can be increased by creating more electroactive sites in electrode materials (i.e., nanoscale and porous electrode materials). In addition, extending the operating voltage (*V*) window can enhance the Es. However, the maximum operating voltage depends on the type of electrolyte used [167]. Even though supercapacitors have higher capacitance than conventional capacitors, they lack specific energy compared to lithium-ion batteries [168].

#### 3.3.3. Specific Power

One of the main advantages of supercapacitor devices is their exceptional power performance. The specific power (Ps) is defined by the rate at which energy may be stored from or delivered to the load, and it is assessed in W/kg. The specific power (Ps) of a supercapacitor is usually calculated as follows [169].

(5) $$\mathrm{Ps}=\frac{{\left(\Delta V\right)}^{2}}{4M\;ESR}\times 1000,$$

where ∆*V* is the voltage window (*V*), *M* is the mass of both electrodes (g), and *ESR* denotes the equivalent series resistance (*Ω*). According to Equation (5), a high Ps could be obtained by maximizing the voltage window but minimizing the *ESR* [169].

#### 3.3.4. Cycle Stability

The prolonged cycle life of supercapacitors is highly desirable for certain applications. However, an excessively long cycle life (e.g., >1 million cycles) complicates its direct measurement. Therefore, the capacitance retention rate, which can be calculated using galvanostatic charge–discharge data, is used to estimate the cycle life of supercapacitors [126,136,170]. A recent report showed that the capacitance retention rate decreases almost linearly with the square root of the number of cycles [171]. Although further endorsements are required to establish this model, this model unraveled the demanding nature of the direct measurement of the cycle life.

### 3.4. Electrochemical Approaches for Supercapacitors

The efficient characterization of the energy storage capability and power performance of the supercapacitors is mainly determined by the three core parameters: total capacitance (CT), operating voltage (Vo), and equivalent series resistance (ESR) [107]. However, other factors such as energy and power densities, time constant, cycling stability, and operating voltage range are essential for developing more effective electrode materials and novel cell designs. These performance-dictating parameters are generally measured by three electrochemical techniques, namely cyclic voltammetry (CV), galvanostatic charge–discharge (GCD), and electrochemical impedance spectroscopy (EIS) [170,172]. The key objective of these techniques is to evaluate the electrochemical properties of the energy storage systems from different viewpoints. Researchers often use two- or three-electrode systems and generally measure three primary parameters: current, voltage, and time, while other necessary parameters are calculated from them. Figure 10d illustrates the schematic of a three-electrode setup consisting of a working electrode, a reference electrode, a counter electrode, and an electrolyte solution (i.e., organic solvent or aqueous solution). Active materials are either coated on the working electrode or used directly as the working electrode; hence, the working electrode is the one that is to be tested. A saturated calomel electrode (SCE) and platinum (Pt) electrode are often employed as reference and counter electrodes, respectively. On the contrary, in a two-electrode system, two current collectors, i.e., a pair of electrodes, are immersed in an electrolyte and kept apart by a porous separator [173]. A two-electrode system provides the specific capacitance of the entire cell, while a three-electrode system gives the specific capacitance of the electrode active material [1,174]. It is worth noting that the outcome of the measurements using different mechanisms may be contradictory; hence, it is crucial to present the obtained results along with the applied parameters [2,175,176].

Relating to the uses of various acids in gel electrolytes for supercapacitors, H_2_SO_4_ and H_3_PO_4_ are often utilized [39]. Apart from improving conductivity, these acids have several important functions. To begin with, they support the development of a stable gel structure. This is essential to preserving the electrolyte’s integrity and avoiding leaks or gradual degradation. Second, by supporting the electrolyte’s pH level optimization, these acids can guarantee the supercapacitor operates at its best. They establish a setting that is favorable to effective charge storage and ion transport. Because of their compatible nature with the other components of the supercapacitor and their chemical properties, H_2_SO_4_ and H_3_PO_4_ are specifically utilized in gel electrolytes. These acids are perfect for this application because they have the desired conductivity and stability properties.

#### 3.4.1. Cyclic Voltammetry (CV)

**Figure 11:**
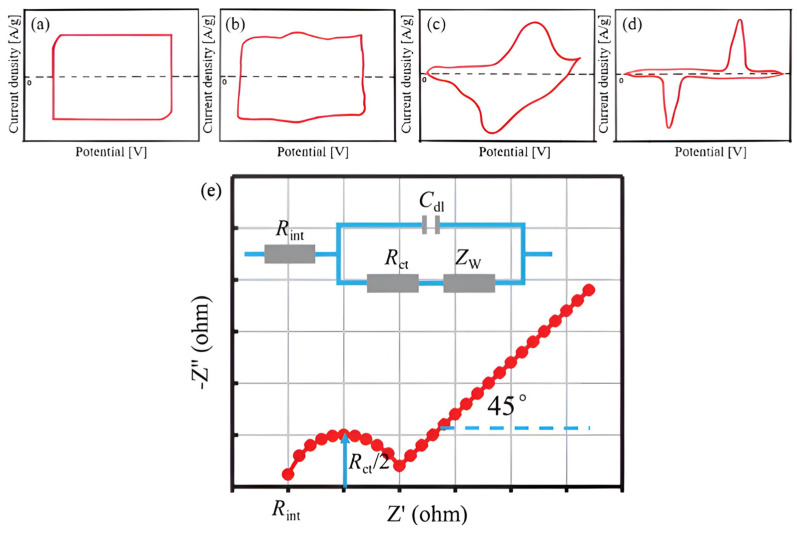
Schematic cyclic voltammograms representing various charge storage mechanisms [107]. (**a**) EDLC, (**b**) surface redox pseudocapacitance, (**c**) Faradaic dominated pseudocapacitance, and (**d**) battery-like behavior. Copyright 2024, MDPI. (**e**) Randles circuit model consisting of internal resistance (Rint), double-layer capacitance (Cdl), charge transfer resistance (Rct), and Warburg impedance (Zw) (in blue) and the corresponding Nyquist plot (in red) [184]. Reproduced with permission from Ref. [184]. Copyright 2019, Wiley.

When the potential is linearly modulated between two different standards (potential window) with time, the electrode current response is determined using cyclic voltammetry (CV) [177]. Therefore, the CV curves (i.e., voltammograms) represent the dependencies of the current on the potential under a fixed scan rate (V/s). CV is the most effective method for elucidating the reversibility and kinetics of charge transfer processes [178]. Figure 11 shows schematic voltammograms for different charge storage mechanisms. The shape of the CV curves makes it possible to differentiate between mechanisms in energy storage devices. For example, EDLCs exhibit a rectangular CV curve (Figure 11a), indicating a lack of redox reactions within the electrochemically stable potential window. On the other hand, pseudocapacitors produce distinct CV curves (Figure 11b–d) that often display sharp redox peaks [103,179,180]. The CV can also be used to assess the electrochemical stability of supercapacitors by performing multiple cycles. Moreover, the specific capacitance of the electrode materials can be evaluated from CV data using the following relation [1]:

(6) $$\mathrm{Cs}=\frac{\int I\left(V\right)dV}{ms\;\left(V_{a}-V_{b}\right)}$$

where *m* is the mass of the electrode used, *s* is the scan rate, ∫I(V)dV is the area enclosed by the curve, and $(V_{a}-V_{b}$) is the potential window [1].

Concerning the CV analysis of gel polymer electrolytes (GPEs) for supercapacitors, Park et al. [181] produced archetypal methacrylate-based GPEs supercapacitors, and they analyzed the electrolytes’ CV. The authors proposed that the working mechanisms of GPEs might improve the rate capability and cycle stability of activated carbon supercapacitors even at 3.4 V by acting as an ion reservoir and having binder-like effects. While the intended GPEs cause a modest increase in bulk and diffusion resistance during the first phase, they act as a binder-like device, preventing the detachment of AC particles and the worsening of impedance metrics when cycling at 3.4 V.

#### 3.4.2. Galvanostatic Charge–Discharge (GCD)

In a galvanostatic charge–discharge (GCD) measurement, subsequent charging–discharging processes can be performed at a constant current value. It measures the electrode voltage as a function of time during the entire charge–discharge procedure. GCD has been recognized as the most versatile and precise method to evaluate several superconductor parameters, including the specific capacitance, specific energy, specific power, potential window, and cycle stability [2,103].

The specific capacitance (Cs) may be calculated from the results of the GCD experiment using the following relation [1,103,122]:

(7) $$\mathrm{Cs}=\frac{I\cdot \Delta t}{m\cdot \Delta V}$$

where *I* is the constant current, ∆*t* is the charging or discharging duration, ∆*V* is the window of the voltage (potential window), and *m* is the mass of the active materials.

#### 3.4.3. Electrochemical Impedance Spectroscopy (EIS)

Electrochemical impedance spectroscopy (EIS) measurements can be employed to evaluate electrical resistance quantitatively. In addition, the characterization of charge transfer, mass transport, and charge storage mechanisms, as well as the assessment of the capacitance, energy, and power properties, have all been performed with this technique [2,111,139,182,183].

During an EIS measurement, a conductive electrode is usually subjected to voltage from an alternating current (AC) source over a wide range of frequencies (10^−2^ to 105 Hz) [139,184]. EIS spectra are usually expressed graphically in both the Bode plot and the Nyquist plot. The most frequently used form is the Nyquist plot, which compares the real part to the imaginary part of impedance at various frequency rates. A typical Nyquist plot, Figure 11e, can be divided into high- and low-frequency regions and may be used to calculate two crucial characteristics: charge transfer resistance (Rct) and equivalent series resistance (ESR) depending on whether the semicircle is created at a 45° or 90° angle [139,185,186].

## 4. Conducting Polymer (CP) Gel-Based Electrode Materials

The supercapacitor performance such as the capacitance and charge storage capability depend on the electrode materials. Thus, searching for an ideal electrode material to improve the supercapacitor performance is always noteworthy. There are certain characteristics of an ideal electrode for supercapacitors including a large surface area, thermal stability, high conductivity, high conductivity, controlled porosity, and relatively low manufacturing cost [187]. To obtain the superb performance of the supercapacitors, researchers are still searching for suitable materials to modify the features of electrodes. Conducting polymers are considered one of the significant electrode materials exploited thus far for supercapacitance purposes [188]. Due to the numerous limitations of other electrode materials like carbon nanomaterials, conducting polymer gels have the potential to overcome such drawbacks with enhanced electrochemical properties [189].

### 4.1. CP Hydrogel Electrode (CPH)

Among the CP hydrogel-based electrodes (CPHs), the preparation of 3D PANI hydrogel with a high specific capacitance value of 750 F/g at a current density value of 1 A/g was reported by Guo et al. [190]. The advantages of using such a self-cross-linked PANI hydrogel as an energy storage material are its charge storage capability and relatively high mechanical strength. The PEDOT-PSS hydrogel electrode [191] was used in supercapacitors by the Inganas group which is considered to be the pioneer in this area. Nonetheless, the electrode exhibited a reduced mechanical potency and lesser specific energy due to the high molecular mass of the PEDOT-PSS components. These drawbacks were overcome by using another conducting polymer, PPy, by depositing it onto the PEDOT-PSS electrode with a high-performance capacitor [39].

Among the CPHs, the most extensively used polymers are poly (3, 4-ethylenedioxythiophene) (PEDOT), PANI, and PPy [192]. From these three materials, PANI provides promising applications in energy storage devices including supercapacitors [190]. PANI in gel forms shows a remarkable capacitance, very good electrical conductivity, and cyclic stability [193]. The freshly prepared PANI gel exhibited an ionic conductivity value of 0.11 S/cm (293 K), and the corresponding electrode reached a large value of Cs (480 F/g at 0.2 A/g) and an excellent capacitance retention in three electrode systems. Moreover, the freshly prepared PPy hydrogel electrode was designated utilizing the PVA-H_2_SO_4_ electrolyte which demonstrated a Cs value of 380 F/g, a larger rate capability, and a maximum areal capacitance value of 6.4 F/cm^2^ [39]. Consequently, it appears that the materials as-fabricated are excellent choices for flexible energy storage devices. In addition, owing to its very good electrochemical performance and superb flexibility, the freshly prepared electrode material offers a new era for the preparation of CPH in energy storage schemes including supercapacitors.

### 4.2. CP-Based Composite Materials

To improve the supercapacitor performance, researchers are introducing various materials such as carbon nanotubes (CNTs), metal oxides (MOs), graphene oxide (GO), and other suitable polymers (for instance, PVA) with conducting polymers (CPs). The incorporation of these materials with CP-based gel or hydrogel can be found in the literature [34,194,195] which can enhance the stability of CP, the mechanical integrity, and ultimately the overall performance of the supercapacitors fabricated using CP-based gel materials. These types of composites are discussed below:

#### PANI-CNT Composites

Nanomaterials fabricated with carbon-based materials exhibit several outstanding merits such as a decent electrical conductivity, great specific surface areas, cyclic strength, and so on. Thus, it is noteworthy to prepare composites by introducing carbon-based nanomaterials including carbon nanotubes (CNTs) with CPs such as PANI. Numerous studies can be found in the literature showing that CP-CNT composites can enhance the electrochemical properties compared to bare CPs [123,196,197], making CNTs suitable materials for CP-based supercapacitors. For instance, CNT/PANI composite hydrogels were synthesized with a capacitance value of 680 mF/cm^2^ at 1 mA/cm^2^ [42]. Other than CNT/PANI composites, PANI/multi-wall CNTs (MWCNTs) composites were prepared by Mi et al. [197] with a specific capacitance value of 322 F/g and a large specific energy density value of 22 W h/kg. These electrochemical values are ~12 times higher in comparison to pure MWCNTs. By introducing MWCNTs with PANI, the higher specific capacitance value of 552.11 F/g at a current density of 4 mA/cm^2^ was reported in Reference [196]. The authors suggested that PANi/MWCNT in a nanostructure form with unique tubular morphology may be prepared on a wide scale using a low-temperature oxidative polymerization approach. Further, PANI/single-wall CNTs (SWCNTs) composites displayed an enhanced specific capacitance value of 236 F/g compared to SWCNTs (23.5 F/g) [198].

### 4.3. PANI Metal Oxides (MO) Composites

The CP gel-based MO composites for supercapacitor application are an exciting area of research for the scientific community. In connection with this, Huang et al. [199] reported PANI-MnO_2_ composite gel materials for supercapacitor application. The prepared composites possessed a 3D porous morphology which facilitates the transmission of electrons. These features made the prepared composites suitable for supercapacitor applications. The electrochemical properties such as the constructed electrode revealed a capacitance (Cs) of 293 F/g relating to a mass loading of 1.5 mg/cm^2^ for the scan rate of 10 mV/s. The Cs value decreases with the increase in mass loading. Therefore, it is possible to speculate that this excellent supercapacitor performance with the freshly prepared composite electrode is due to their conductive framework and the porous gel structure. PANI/MoS_2_ nanocomposites were prepared using a hydrothermal redox reaction process and attained the large specific capacitance value of 450 F/g, and it was possible to improve the cycle stability with 80% retention of the primary value after 2000 charge–discharge progressions [200]. PANI/MoS_2_ nanocomposites were developed, providing greater electrode/electrolyte integration [201]. The attained electrode was suitable for a specific capacitance value of 669 F/g through a voltage window of ±0.6 V for a regular current density value of 1 A/g. When the voltage window increased to 0.8 and ±1.0 V, the capacitance also increased, remaining at values of 821 and 853 F/g, respectively. The authors noted that the distinct topologies of the prepared PANI/MoS_2_ composites reduced the diffusion channels for both electrons and ions and provided double electrochemically active components for improved electrode/electrolyte interaction. In addition, the copolymer derived from PANI and PPy by Rahman Khan et al. [39] showed the highest capacitance value of 300 F/g, an energy density value of 23 Wh/kg, and a power density value of 900 W/kg. In this case, the prepared copolymer was mixed with (NH_4_)_2_S_2_O_8_ and CoO; then, the blend was polished on the current collector. On the other hand, the assortment comprising activated charcoal and electrolytes was attached on an alternative current collector (Figure S1a). Four cells were prepared and joined in sequences for a single setup (Figure S1b). The experimental setup and supercapacitor cell prepared using such copolymer materials are presented in Figure S1 [39]. The authors suggested that such types of composites are promising in the area of hybrid supercapacitors.

### 4.4. PANI Graphene Oxide (GO) Composites

PANI-GO composites are a significant class of materials for supercapacitor purposes. For example, the preparation of PANI-GO or PANI-reduced GO (rGO) composites was reported for improved supercapacitor performance [202,203]. PANI/Graphene (GN) composites were synthesized containing GO in acid in Reference [204]. The obtained composites displayed a higher specific capacitance value of 480 F/g for a current density of 0.1 A/g. The authors also suggested that in the doping of PANI with GN or GO, the prepared materials have much higher specific capacitance and well-cycling stabilities. In this context, Wang et al. [205] fabricated PANI/rGN composites to investigate the electrochemical capacity. They reported that the specific capacitance can attain the highest value of 740 F/g with a current density is 0.5 A/g, and the retention of the primary capacitance value was 87% following charge/discharge for 1000 cycles, for a current density of 10 A/g. Such 3D-type composite structures can offer large interfacial areas, narrow ion diffusion routes, and very fast electrical routes which make them advantageous for supercapacitor applications.

Thus, it is known that GO is a valuable GN derivative, and researchers have performed numerous studies on CPs/GO. The highly efficient electrode material was fabricated using PANI doped with GO [203]. The obtained nanocomposites showed a high conductivity value of 10 S/cm for 22 °C and an improved specific capacitance value of 531 F/g in possible windows ranging from 0 to 0.45 V at 0.2 A/g, better than PANI alone (216 F/g). Such data indicate that the incorporation of GO has a potential impact on PANI. Sun et al. [202] synthesized 3D PANI/rGO nanocomposites which possessed a good specific capacitance of 701 F/g using a current density of 1 A/g. They can also maintain 92% of the preliminary value for 1000 cycles. The prepared composites exhibited the merits of both PANI and rGO.

In addition, the scientists also prepared CP-based gels or hydrogels of ternary composites, which possessed a unique type of nanomaterial for better supercapacitive ability. For instance, Jayakumar et al. [206] fabricated 3D GN/PANI/MnO_x_ composites and investigated the electrochemical properties in a three-electrode system. Such composite hydrogels can supply a great Cs value of 955 F/g for 1 A/g and capacitive retention of 89% for 1000 cycles. The capacitive retention was 69.1% after 5000 successive cycles with a current density of 20 A/g. The observed superb electrochemical enactment of the prepared composites was because of the well-organized nanostructure and interactive effects of the reactant from which the composite was prepared. Mondal et al. [207] fabricated ternary composites of rGO/Fe_3_O_4_/PANI for a binder-free supercapacitor. The attained electrochemical outcomes were a Cs of 283.4 F/g, power density (Ps) of 550 W/kg, and energy density (Es) of 47.7 Wh/kg for a current density of 1 A/g.

### 4.5. PPy-CNT

Among CPs, PPy is one of the CPs with the utmost potential owing to its numerous merits including its electrochemical properties, simple synthesis, low cost, and good conducting nature and it has been applied in different fields like supercapacitors [208]. However, PPy possesses less cyclic stability. To improve the cyclic stability of PPy, researchers have focused on studies of PPy-based CNTs for supercapacitor application. For example, Tang et al. [209] fabricated PPy-based CNT composites for supercapacitors where CNT@PPy was applied as an anode material. In the case of CNT/PPy film, the specific area-based capacitance can reach up to 637 mF/cm^2^ using an electro-deposition time of 2000 s with a current density value of 1 mA/cm^2^. The fabricated supercapacitor exhibited a high energy density value of 40 W h/kg for a power density of 519 kW/kg.

In addition, using CNTs as templates for the fabrication of composite electrodes can enhance the electrochemical effectiveness such as the cycle stability of PPy-based electrodes due to the synergistic effects [210]. Furthermore, SWCNTs were used to fabricate PPy/SWCNTs composite electrodes with a specific capacitance value of 265 F/g, which is better than PPy and SWCNT. The authors indicate the better enactment of PPy/SWCNTs composites owing to the homogeneously treated PPy on SWCNTs which enhanced the functional sites on the PPy structure. MWCNT/PPy composites were prepared by Song et al. [211] with more organized chain packing and a higher molecular conformation which were able to facilitate the attainment of a better electrical conductivity of the composites. Wang et al. [212] fabricated PPy/MWCNT composites where the electrical conductivity was varied with the content of MWCNT loadings. The PPy/bonded CNT composites were obtained by Yang et al. [210] with an improved thermal stability and conductivity of the composite materials. The authors showed the better electrochemical features of the prepared PPy/plasma-activated CNT compared to PPy/CNT composites. The explanation of the better performance of such materials is that the active sites of the nitrogen groups in the PPy ring offer the integration of CNTs with PPy. Both short and long CNTs were introduced to prepare PPy/PSS-CNT for the first time by Zhou et al. [213], where the capacitive features of electrodes based on PPy were notably enhanced for the extent of both CNTs. It was mentioned that owing to the porous surface morphology and core-shell nanostructure of long CNTs, the supercapacitor performance as well as the cyclic stability of the PPy/PSS electrode were considerably enhanced. In another study, a “hybrid supercapacitor” electrode depending on PPy/CNT composites was noticed [214]. They showed that fabricated supercapacitors are able to retain a 92% capacitance for over 3000 cycles, demonstrating a superb cyclic strength.

### 4.6. PPy-GO Composites

It is known that GO and rGO can be derived from graphene (GN) [215,216]. These materials can improve the supercapacitor enactments when they are integrated with PPy. For example, Zhu et al. [217] fabricated composites of PPy-based GN with controlled and distinct morphological features showing a superb electrochemical ability. The 3D core-shell structure of GO/PPy was prepared by Wu et al. [218] demonstrating a remarkable specific capacitance and tremendous cycle stability. The specific capacitance value was 370 F/g, with a current density of 0.5 A/g, a mass loading of 8.0 mg/cm^2^, and a capacitance retention of 91.2% for 4000 cycles because of the collaborative impact of both PPy and GO. CP/rGO nanocomposites with different extents of loading of PEDOT, PANI, and PPy were synthesized in Reference [216]. The PPy/rGO composites showed a specific capacitance of 248 F/g and a current density of 0.3 A/g, which is far superior compared to rGO/PEDOT (108 F/g). However, the supercapacitor enactments are poorer than for rGO/PANI, although both the PPy and PANI-based composites were prepared in the same experimental environments. On the other side, to improve the energy storage capability of PPy-based electrodes, scientists have prepared hybrid composites comprising two different types of electroactive substances like PPy and metal oxides. For instance, Shayeh et al. [219] fabricated a PPy/MnO_2_ nanowire with a specific capacitance value of 203 F/g which is two times better than bare PPy (109 F/g). Such excellent electrochemical performances of the prepared composites are owing to the synergistic impacts enabling by both PPy and MnO_2_.

Therefore, it can be said that freshly-fabricated supercapacitors from the CP gel-based materials with a better specific capacitance, outstanding rate capability, and good cycling stability can provide prospective materials for forthcoming energy storage applications. However, the proper design and synthesis of appropriate electrode materials like CPs is required for the further development of their electrochemical performance in supercapacitors. In that case, CPGs can provide more advantages in the area of supercapacitors for large applications providing a good electrochemical performance.

## 5. Conclusions and Future Perspectives

CPGs are an exceptional type of nanoarchitecture material and exhibit the merits of superhydrophilicity, biocompatibility, and the combined features of organic conductors and polymers. Also, they have demonstrated several benefits of natural gel materials, such as a very good flexibility and better electrochemical properties. In connection with this, CPGs are effective materials for supercapacitor application which provide excellent promise to meet the demand of the latest electronic devices. Although substantial progress has already been made on CPGs-based materials, there are still promises and possibilities to enhance the supercapacitor performance for large-scale applications. Numerous limitations including the morphological complexity, thermal properties enhancement, and device configuration are still challenges affecting supercapacitor applications. Thus, more efforts can be provided to the CPGs, focusing on their simple functionalization with tunable morphological features and thermal stability, as well as their design and easy fabrication for energy storage systems like supercapacitors. In future work, the properties of CPGs can be altered by incorporating functional groups with the polymers and/or using co-polymerization techniques with the promising polymers to improve their electrochemical properties. Further, advanced characterization and interpretations are needed to accurately illustrate the CPG materials including the surface behavior of the gel matrix and integrated functional materials. Therefore, it is necessary to fine-tune CPG materials focusing on particular properties for a better supercapacitor performance. Through substantial research efforts, supercapacitors fabricated using CPG materials are anticipated to provide a unique possibility in areas of modern energy like renewable energy and energy-based technology which are commercially feasible. Furthermore, the economical manufacturing of electrode materials and devices is highly expected for the large-scale applications of supercapacitors and more technological enrichments.

## Supplementary Materials

The following supporting information can be downloaded at https://www.mdpi.com/article/10.3390/gels10090553/s1, Figure S1: (a) Supercapacitor cell preparation; (b) Experimental setup, where the constructed four cells were coupled in series for a single setup. Reprinted with permission from Reference [38]. Copyright 2022, Elsevier.
