# Supplementary material for: Conducting Polymer-Based Gel Materials: Synthesis, Morphology, Thermal Properties, and Applications in Supercapacitors
Source: Gels. 2024 Aug 26;10(9):553. doi: 10.3390/gels10090553 (PMC11431190; doi:10.3390/gels10090553)
Supplement: Supplementary file 1 [file gels-10-00553-s001.zip › gels-3140289-supplementary.pdf]

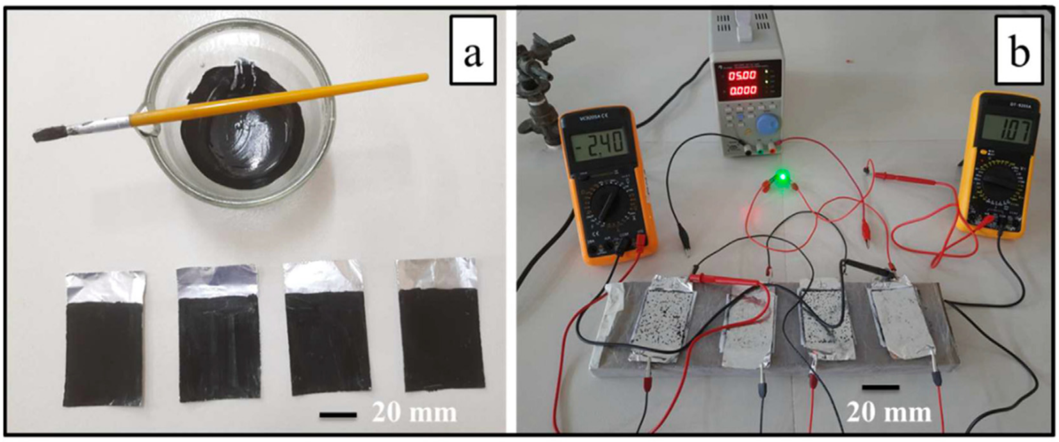

Figure S1. (a) Supercapacitor cell preparation, (b) Experimental set-up, where the constructed four cells were coupled in series for single setup. Reprinted with permission from reference [38]. Copyright 2022, Elsevier.
